# Supplementary material for: Genomic study of taste perception genes in African Americans reveals SNPs linked to Alzheimer’s disease
Source: Sci Rep. 2024 Sep 16;14:21560. doi: 10.1038/s41598-024-71669-9 (PMC11405524; doi:10.1038/s41598-024-71669-9)

Title: Distribution of the raw expression (read counts) of the mRNAs investigated or relevant for the analysis. The numbers plotted are available in Supplemental Table T1E

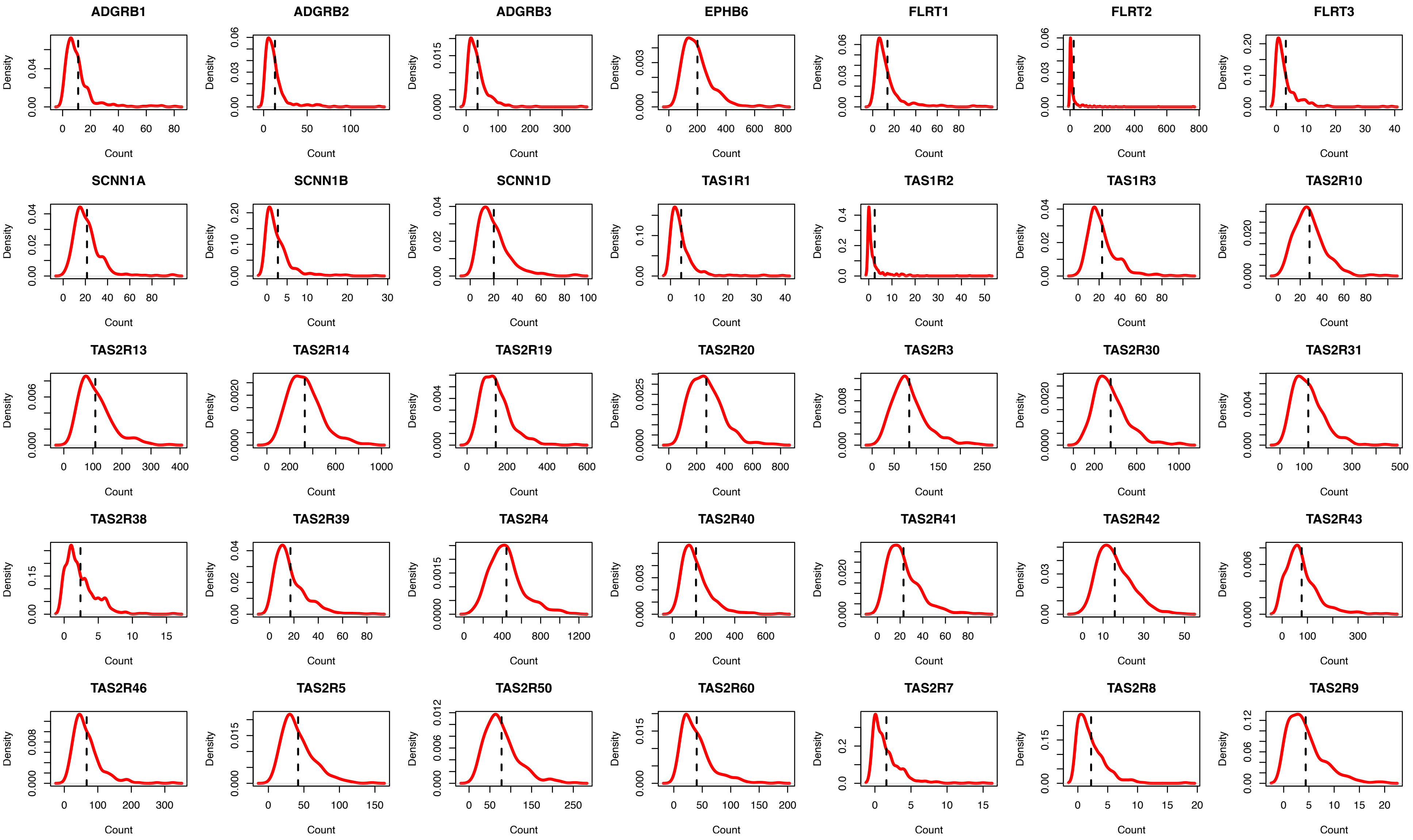

Supplement: Supplementary file 1 — Supplementary Information. [file 41598_2024_71669_MOESM1_ESM.pdf]
